# Association Between a Ban on Smoking in a Hospital and the In-Hospital Onset of Acute Myocardial Infarction

**DOI:** 10.14740/cr404e

**Published:** 2015-06-11

**Authors:** Natsumi Morito, Shin-ichiro Miura, Masaya Yano, Yuka Hitaka, Hiroaki Nishikawa, Keijiro Saku

**Affiliations:** a Department of Cardiology, Fukuoka University School of Medicine, Fukuoka, Japan; b Department of Molecular Cardiovascular Therapeutics, Fukuoka University School of Medicine, Fukuoka, Japan

**Keywords:** Smoking ban, In-hospital onset, Acute myocardial infarction, Direct smoking, Passive smoking

## Abstract

**Background:**

Smoking is an important cardiovascular risk. We hypothesized that a ban on smoking in a hospital could decrease the in-hospital onset of acute myocardial infarction (AMI).

**Methods:**

Our hospital provided separate facilities for smokers and nonsmokers from 1981 to 2002. From 2002 to 2006, we began to introduce smoke-free zones throughout the entire building. During this period, smoking areas and smoking tables were abolished, until the entire hospital became a non-smoking area in 2007. We registered patients who experienced an in-hospital onset of AMI from January 2002 to June 2014. Patients with an in-hospital onset of AMI were defined as those who had AMI but were not under the care of the Departments of Cardiology or Emergency. We observed 25 patients (males/females, 16/9; average age, 70 years) with an in-hospital onset of AMI from 2002 to 2014.

**Results:**

The incidence of in-hospital AMI significantly decreased as the stages of non-smoking areas progressed (P for trend 0.010). Six of the 25 patients died after AMI. The death group showed significantly higher serum levels of peak creatine kinase and lower levels of hemoglobin. In addition, 10 of the 25 patients developed in-hospital AMI after surgery. Anti-coagulant therapy was canceled before an operation in three patients. After an operation, advanced anemia was seen in four patients. In addition, there were no differences in the patient characteristics between the smoking and non-smoking groups except for dyslipidemia.

**Conclusion:**

The spread of a non-smoking policy significantly decreased the in-hospital onset of AMI in our hospital, which suggests that not only direct smoking but also passive smoking is important target for reducing in-hospital AMI.

## Introduction

Smoking is a cause of cardiovascular diseases [1-3]. Thus, non-smoking is important for preventing several lifestyle-related diseases. Both the number of cigarettes smoked daily and the number of pack-years of exposure seem to be associated with the development of impaired fasting glucose and type 2 diabetes [4] and exposure to smoke is associated with the metabolic syndrome [5]. Passive smoking, or second-hand smoking, refers to breathing other people’s tobacco smoke, and causes the same problems as direct smoking [6]. Smoking bans are public policies that prohibit smoking in workplaces and public spaces [7-10]. A reduced incidence of admissions for acute myocardial infarction (AMI) has been associated with a public smoking ban [7]. In addition, admission rates for AMI were reduced as a result of comprehensive smoking bans [8], and a public ordinance that reduced exposure to passive smoking was associated with a decrease in AMI hospitalizations [9]. Thus, smoking bans significantly decrease AMI hospitalizations. Recently, although there are a growing number of restrictions on smoking in hospitals, it is not known whether such restrictions are effective. Thus, both direct smoking and passive smoking are important. Therefore, we hypothesized that a ban on smoking in a hospital to help reduce passive smoking could decrease the in-hospital onset of AMI, and examined the association between an increase in non-smoking areas in a hospital and the in-hospital onset of AMI.

## Methods

### Subjects

Our hospital has been instituting non-smoking policies since 1981. From 1981 to 2002, our hospital provided separate facilities for smokers and nonsmokers. In the next stage, from 2003 to 2006, we began to introduce non-smoking areas throughout the building. Smoking areas and smoking tables were abolished, until smoking was banned throughout the entire hospital in 2007. We registered patients with an in-hospital onset of AMI using the database of Fukuoka University Hospital from January 2002 to June 2014. The in-hospital onset of AMI was defined as patients who had AMI, except for those who were under the care of the Departments of Cardiology or Emergency. We excluded the onset of unstable angina pectoris. Our protocol was approved by the Ethics Committee of Fukuoka University Hospital. We retrospectively collected and analyzed all data after receiving permission.

### Patient characteristics and biochemical parameters

The patient characteristics, including age, gender, body mass index (BMI), smoking state, history of hypertension (HTN), dyslipidemia (DL), diabetes mellitus (DM), and medication use, were obtained from medical records. Patients who had a current SBP/DBP ≥ 140/90 mm Hg or who were receiving antihypertensive therapy were considered to have HTN. Patients with low-density lipoprotein cholesterol ≥ 140 mg/dL and/or triglycerides ≥ 150 mg/dL or high-density lipoprotein cholesterol < 40 mg/dL, or who were receiving lipid-lowering therapy were defined as DL. DM was defined using the Japanese Diabetes Society criteria. Data regarding coronary angiography (CAG), blood pressure (BP), and serum levels of biochemical parameters, such as hemoglobin (Hb), creatinine (Cr), total cholesterol (TC), triglycerides (TG), hemoglobin A1c (HbA1c) and creatine kinase (CK), were also collected.

### Statistical analysis

Statistical analysis was performed using the Stat View statistical software package (Stat View 5; SAS Institute Inc., Cary, NC, USA) at Fukuoka University (Fukuoka, Japan). Data are shown as the mean ± standard deviation (SD). Categorical variables were compared between the groups by a Chi-square analysis. The significance of differences between mean values was evaluated by an unpaired *t*-test or one-way analysis of variance, as appropriate. A value of P < 0.05 was considered significant.

## Results

### Patient characteristics

We observed 25 patients (males/females, 16/9; average age, 70 years) with an in-hospital onset of AMI from 2002 to 2014 (Table 1). The percentages of HTN, DM, DL and smoking were 48%, 48%, 20% and 56%, respectively. The percentages of significant coronary stenosis of the right coronary artery (RCA), left anterior descending artery (LAD) including the left main trunk (LMT) and circumflex artery (LCX) were 20%, 56%, and 11%, respectively. Seventeen patients (68%) underwent percutaneous coronary intervention (PCI), and the peak CK was 2,848 IU/dL.

**Table 1:** Patient Characteristics in All Patients

Patients, n	25	Anticoagulant, n (%)	8 (32)
Age, years	70 ± 9	Hb, g/dL	11.1 ± 2.9
Male, n (%)	16 (64)	Cr, mg/dL	0.9 ± 0.3
Post-operation, n (%)	10 (40)	eGFR, mL/min/1.73m^2^	69 ± 25
Death, n (%)	6 (24)	TC, mg/dL	189 ± 53
HTN, n (%)	12 (48)	TG, mg/dL	108 ± 60
DM, n (%)	12 (48)	HbA1c, %	7.5 ± 1.8
DL, n (%)	5 (20)	SBP, mm Hg	130 ± 21
IHD p/s PCI, n (%)	7 (26)	DBP, mm Hg	73 ± 12
Smoking, n (%)	15 (56)	Target vessels	
Medication		LAD or LMT/LCX/RCA, n (%)	15 (56)/5 (20)/3 (11)
CCB, n (%)	6 (22)	PCI, n (%)	17 (68)
ACE-I/ARB, n (%)	6 (22)	Peak CK, IU/L	2848 ± 2550
β blocker, n (%)	1 (4)		
Statin, n (%)	4 (15)		

Continuous variables are expressed as mean ± SD. HTN: hypertension; DM: diabetes mellitus; DL: dyslipidemia; IHD: ischemic heart disease; PCI: percutaneous coronary intervention; CCB: calcium channel blocker; ACE-I: angiotensin converting enzyme; ARB: angiotensin II receptor blocker; Hb: hemoglobin; Cr: creatinine; eGFR: estimated glomerular filtration rate; TC: total cholesterol; TG: triglycerides; HbA1c: hemoglobin A1c; SBP: systolic blood pressure; DBP: diastolic blood pressure; LAD: left anterior descending artery; LMT: left main trunk; RCA: right coronary artery; LCX: left circumflex artery; CK: creatine kinase.

### Association between non-smoking areas in a hospital and the in-hospital onset of AMI

**Figure 1:**
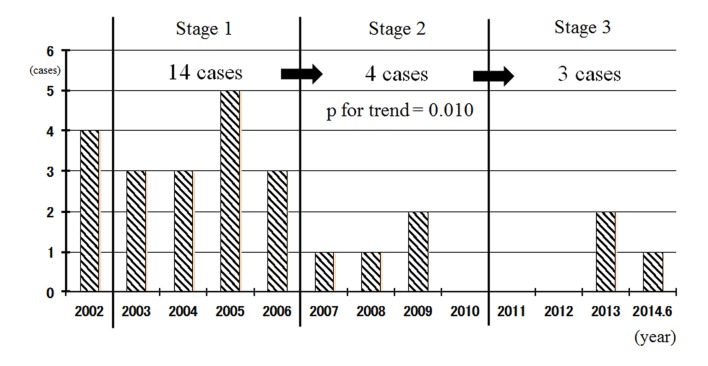
Association between non-smoking areas in a hospital and the in-hospital onset of AMI. Non-smoking areas throughout the building (stage 1, 2003 - 2006), smoking was banned throughout the entire hospital at the early (stage 2, 2007 - 2010) and late (stage 3, 2011 - 2014) phases.

We divided the study period into three stages: 2003 - 2006 (stage 1), 2007 - 2010 (stage 2) and 2011 - 2014 (stage 3) (Fig. 1). The incidence of in-hospital AMI significantly decreased as the stages progressed (P for trend 0.010).

### Comparison of patient characteristics of the survival and death groups after AMI

Six of the 25 patients died after AMI (Table 2). We analyzed the differences between the survival and death groups. The death group showed significantly higher maximum CK and lower Hb levels.

**Table 2:** Comparison of Patient Characteristics of the Survival and Death Groups After AMI

Variables	Death	Survival	Death vs. survival P value
Patients, n	6	19	
Age, years	76 ± 7	68 ± 9	0.10
Days	10 ± 8	23 ± 29	0.29
Peak CK, IU/L	5,768 ± 2,234	1,954 ± 1,855	0.0003
BMI, kg/m^2^	23 ± 2	22 ± 3	0.44
SBP, mm Hg	139 ± 22	127 ± 20	0.23
DBP, mm Hg	74 ± 12	72 ± 12	0.81
Hb, g/dL	8.7 ± 2.9	11.8 ± 2.5	0.002
eGFR, mL/min/1.73 m^2^	64 ± 35	70 ± 22	0.60
HTN, n (%)	3 (50)	9 (47)	0.91
DM, n (%)	3 (50)	9 (47)	0.92
DL, n (%)	1 (17)	4 (21)	0.82
Smoking, n (%)	3 (50)	12 (63)	0.32
Operation, n (%)	3 (50)	8 (42)	0.58

Days: the days from hospitalization to onset of AMI; CK: creatine kinase; BMI: body mass index; SBP: systolic blood pressure; DBP: diastolic blood pressure; Hb: hemoglobin; eGFR: estimated glomerular filtration rate; HTN: hypertension; DM: diabetes mellitus; DL: dyslipidemia.

### Comparison of patient characteristics of operation and non-operation groups

Ten of the 25 patients (40%) developed in-hospital AMI after a surgical operation (Table 3). There were no differences in the patient characteristics between the operation and non-operation groups except for DL. The onset occurred within 1 or 2 days after the operation in five cases. Anti-coagulant therapy was canceled before the operation in three patients. After the operation, advanced anemia was seen in four patients.

**Table 3:** Comparison of Patient Characteristics of the Operation and Non-Operation Groups

Variables	Operation	Non-operation	Operation vs. non-operation P value
Patients, n	10	15	
Age, years	72 ± 11	69 ± 8	0.43
Days	23 ± 31	17 ± 22	0.59
Peak CK, IU/L	3,060 ± 3,100	2,697 ± 2,183	0.73
BMI, kg/m^2^	23 ± 3	21 ± 3	0.08
SBP, mm Hg	130 ± 23	129 ± 19	0.91
DBP, mm Hg	72 ± 15	74 ± 9	0.73
Hb, g/dL	11.6 ± 1.9	10.6 ± 3.4	0.40
eGFR, mL/min/1.73 m^2^	63 ± 19	72 ± 28	0.37
HTN, n (%)	6 (60)	6 (40)	0.35
DM, n (%)	6 (60)	6 (40)	0.35
DL, n (%)	4 (40)	1 (7)	0.04
Smoking, n (%)	6 (60)	9 (60)	0.82

All abbreviations as in Table 2.

### Comparison of patient characteristics of the smoking and non-smoking groups

Finally, we examined the differences in patient characteristics between the smoking and non-smoking groups (Table 4). There were no differences between the smoking and non-smoking groups except for DL.

**Table 4:** Comparison of Patient Characteristics of the Smoking and Non-Smoking Groups

	Smoking	Non-smoking	Smoking vs. non-smoking P value
Patients, n	15	10	
Age, years	69 ± 9	72 ± 9	0.52
Days	24 ± 31	12.5 ± 13.0	0.28
Peak CK, IU/L	2,989 ± 2,720	2,649 ± 2,411	0.75
BMI, kg/m^2^	23 ± 3	22 ± 3	0.79
SBP, mm Hg	128 ± 18	133 ± 25	0.57
DBP, mm Hg	71 ± 12	75 ± 14	0.40
Hb, g/dL	11.6 ± 3.0	10.1 ± 2.4	0.25
eGFR, mL/min/1.73 m^2^	73 ± 21	60 ± 29	0.24
HTN, n (%)	7 (47)	5 (50)	0.87
DM, n (%)	7 (47)	5 (50)	0.87
DL, n (%)	5 (33)	0 (0)	0.04
Operation, n (%)	6 (40)	4 (40)	1.00
Death, n (%)	3 (20)	3 (30)	0.59

All abbreviations as in Table 2.

## Discussion

In this study, we assessed the association between non-smoking areas in a hospital and the in-hospital onset of AMI. First, we found that the incidence of the in-hospital onset of AMI significantly decreased as the number of non-smoking areas increased. Second, the patients who died after AMI showed significantly higher serum levels of maximum CK and lower levels of Hb. In addition, anti-coagulant therapy was cancelled before an operation in three patients, and advanced anemia was seen after an operation in four patients.

The most interesting finding was that the incidence of in-hospital AMI significantly decreased with the spread of non-smoking areas. Passive smoking is associated with a risk of coronary heart disease [11-13]. The implementation of non-smoking legislation has been associated with significant reductions in both hospital admissions attributable to cardiovascular conditions and those attributable to respiratory conditions [11]. In addition, passive smoking is associated with an increase in the risk of coronary heart disease (CHD), and the public health consequences of passive smoking with regard to CHD may be important [12]. In a prospective cohort study, regular exposure to passive smoking at home or work was shown to increase the risk of CHD among non-smoking women [13]. Smoking bans to prevent passive smoking significantly decreased AMI hospitalizations [7-10]. Thus, passive smoking can have important effects in public areas, as well as at home and work. In this study, we confirmed that passive smoking is also critical in a hospital setting. Since there were no differences in the patient characteristics between the smoking and non-smoking groups except for DL, the elimination of both direct smoking and passive smoking may be important for reducing the in-hospital onset of AMI.

Although the elimination of both direct and passive smoking offers the best strategy for preventing the in-hospital onset of AMI, the present study raises some important points for the prevention of AMI. Since the patients who died after AMI showed significantly lower levels of Hb, and since advanced anemia was seen after an operation in four patients, we should prevent blood loss as much as possible and recover anemia before an operation. In addition, since anti-coagulant therapy was cancelled before an operation in three patients, optimal heparinization during an operation may be needed to prevent AMI.

In conclusion, a non-smoking program significantly decreased the in-hospital onset of AMI in our hospital, which suggests that both direct smoking and passive smoking are important for decreasing in-hospital AMI.
